# Serum androgen profile of elite athletes

**DOI:** 10.1210/clinem/dgag196

**Published:** 2026-05-07

**Authors:** David J Handelsman, Stephane Bermon

**Affiliations:** ANZAC Research Institute, University of Sydney, Sydney, NSW 2139, Australia; Andrology Department, Concord Hospital, Sydney, NSW 2139, Australia; Health and Science Department, World Athletics, 6-8 quai Antoine 1er, 98000 Monaco, Monaco; LAMHESS, Université Côte D'Azur, Nice, France

**Keywords:** testosterone, dihydrotestosterone (DHT), androstenedione (A4), T/DHT ratio, T/A4 ratio, steroid liquid chromatography–mass spectrometry

## Abstract

**Introduction:**

The binary sex classification protects elite female athletes from the male physical advantages arising from puberty; however, mutations causing XY disorders of sex development (DSD) challenge the binary sex classification. Hormonal features of the most frequent XY DSDs in sports, androgen insensitivity syndrome (AIS) and 5α-reductase type 2 deficiency (5ARD) are not well described.

**Aims:**

To define serum androgen profiles in elite athletics (track and field) events

**Methods:**

Serum samples from elite athletes (n = 1689; male 889, female 800; n = 5516 samples, median 3 per person) were analyzed by liquid chromatography–mass spectrometry (LCMS) for serum testosterone (T), dihydrotestosterone (DHT), and androstenedione in 19 World Anti-Doping Agency (WADA)-accredited laboratories. Serum steroids and ratios were analyzed by linear mixed model for repeated measures with adjustment for multiple comparisons and imputation for left-censored (dihydrotestosterone [DHT]) data. Among females, XY DSD identified by adult male serum T concentrations, had clinical diagnosis of 5ARD, AIS, or unclassified (UNCL).

**Results:**

XY DSD comprised 12 individuals with 5ARD with 2 with AIS (1 complete). Excluding pregnancy or androgen doping samples, men had higher serum T (n = 2671, 19.7 ± 0.1 nmol/L) and T/DHT ratio (n = 1903, 14.4 ± 0.2) than women (n = 2793, 1.1 ± 0.1 nmol/L; n = 1809, 3.7 ± 0.12, respectively) with XY DSD featuring male-like results (n = 52, 28.9 ± 0.8 nmol/L; n = 39; 32.0 ± 1.3).

**Conclusion:**

The prevalence of XY DSD in elite female athletes greatly exceeds that of the community with a high proportion of androgen-sensitive 5ARD. Serum T/DHT ratio by LCMS to characterize 5ARD can be extended to elite athletes.

Almost all sports operate with a traditional binary sex classification. For power sports, those in which strength, speed, and/or endurance are crucial to success, the binary sex classification is indispensable to protect female competitors against insuperable male physical advantages arising from male puberty (1-4). These male physical advantages arising from male puberty are due to the sustained 20- to 30-fold increase in testosterone (T) production, which creates larger and stronger muscles and bones and higher cardiorespiratory capacity and hemoglobin, among other sex differences (2). Androgen effects of lesser magnitude may also be due to androgen imprinting from the exposure during fetal male sexual differentiation as well as of boys, but not girls, to adult circulating T concentrations during minipuberty (5), the first 6 months of neonatal life. Genetic physical (dis)advantages for sports, such as mutations in α-actinin-3 (6) or erythropoietin receptor (7) and others leading to differences in bone structures (eg, limb length, height) or muscle function are acceptable components of an athlete's natural talents (8) and occur indiscriminately in men and women. By contrast, genetic mutations causing XY disorders of sex development (DSD) can create male-bodied athletes with female gender identity. That is, these individuals have XY rather than XX chromosomes, testes instead of ovaries and uterus, and adult male rather than female circulating T; however, based on their female gender identity may seek to compete in elite female events (9). Among XY DSDs, androgen-insensitive individuals with inactivating mutations of the androgen receptor (androgen insensitivity syndrome [AIS]) or complete gonadal dysgenesis (including Swyer syndrome), do not experience androgenizing effects of male puberty and therefore remain eligible to compete in elite female events. By contrast, androgen-sensitive XY DSDs are not eligible according to World Athletics eligibility regulations. The most frequent XY DSDs are 5α-reductase type 2 deficiency (5ARD) and AIS (10-12), but their hormonal features among elite athletes are not well described. Therefore, this study aimed to define the serum androgen profiles of individuals participating in elite athletic (track and field) events provided by liquid chromatography–mass spectrometry (LCMS) analysis of serum androgen profiles collected during routine antidoping tests in World Anti-Doping Agency (WADA)-accredited laboratories.

## Materials and methods

Serum samples collected between June 2018 and August 2025 (prior to new SRY eligibility regulation (13)) from high performing elite athletes on the Athletics Integrity Unit (AIU) Registered Testing Pool (RTP) (14), currently (over 600 athletes) or previously, were obtained from routine urine and/or blood antidoping tests. All serum samples that underwent blood steroid profiling were included in this study without further selection. No blood or urine tests were performed for screening of sex/gender eligibility.

Serum samples underwent analysis by LCMS in 19 WADA-accredited antidoping laboratories. WADA accreditation requires ongoing compliance with governance regulations for antidoping laboratories (15), including participation in the WADA EQAS scheme (16). For data analysis, an athlete's serum sample results were de-identified with a unique code and the steroid results together with age and analytical laboratory, but no other identifying information, were provided and analyzed without any knowledge of the individuals with XY DSD diagnoses.

Athletes in the RTP self-identified as male and female. When a female athlete returned a serum T concentration within the male range, which could not be explained by an antidoping rule violation, they were classified as a presumptive XY DSD. This activated a standard operating procedure for confidential data sharing between the Athletics Integrity Unit and the World Athletics Medical Manager (S.B.). When a final clinical genetic diagnosis of the XY DSD, provided by the athlete's own medical team, was known to the Medical Manager it was associated with the code for that athlete. Therefore, the results were classified as Male, Female, 5ARD, or AIS (including complete and partial) or unclassified (UNCL), the latter being when serum T was in the male range (without antidoping rule violation) but the final clinical diagnosis remained unknown to the Medical Manager when the athlete did not know or pass to World Athletics the final diagnosis.

The serum androgen profile comprised measurement of serum testosterone (T), dihydrotestosterone (DHT), and androstenedione (A4) and their ratios (T/DHT, T/A4).

Reference ranges for male serum T, DHT, and T/DHT ratio were derived from the Raine birth cohort study for 618 men at age 20 years which measured serum T and DHT in 618 men (17) by steroid LCMS profiling at the ANZAC Research Institute Andrology laboratory (18, 19).

Serum steroid concentrations and ratios were analyzed by linear mixed model for repeated measures with Bonferroni adjustment for multiple comparisons and data imputation for left-censored (DHT) data (20). For most efficient use of the data, the unit of analysis was the serum sample (representative of the diagnostic category) rather than the athlete. This was consistent with a repeated measures analysis (in mixed linear model) showing no bias with any significant trends with visit number for serum T, DHT, and T/DHT and T/A4 ratio.

As the T/DHT ratio data displayed significant multimodality, these data were further analyzed to separate the component peaks as a Gaussian mixture population using the mixtools program in R (21). This decomposed the overall T/DHT ratio data into 3 peaks with estimates of their mean T/DHT ratio and the prevalence of each subpopulation. The goodness of fit between multimodalities was evaluated by the Bayesian Information Criterion and the presence of multimodality (vs unimodality) by Hartigan's dip test (22).

## Results

Serum samples were obtained from the RTP database comprising the highest performing athletes among the elite competitions (14). All serum samples without restriction were used in this study. Athletes from the RTP (n = 1689) provided 5516 serum samples (median 3 per person) samples. These samples included 195 (3.5%) in competition and 5321 (96.5%) out of competition, with the proportions not differing between diagnosis categories (*P* > .6).

These athletes self-identified as male (n = 889) or female (n = 800) with the females including 22 XY DSD diagnoses, comprising 12 with 5ARD, 2 with AIS (1 complete, 1 partial), and 8 unclassified (UNCL) (Table 1, Fig. 1). The prevalence of 5ARD among XY DSD athletes was 6 (1.5-24) times that of AIS or 12 (1.7-85) times that of complete AIS (CAIS) (both *P* < .001, Fisher's test), omitting the UNCL category.

**Figure 1:**
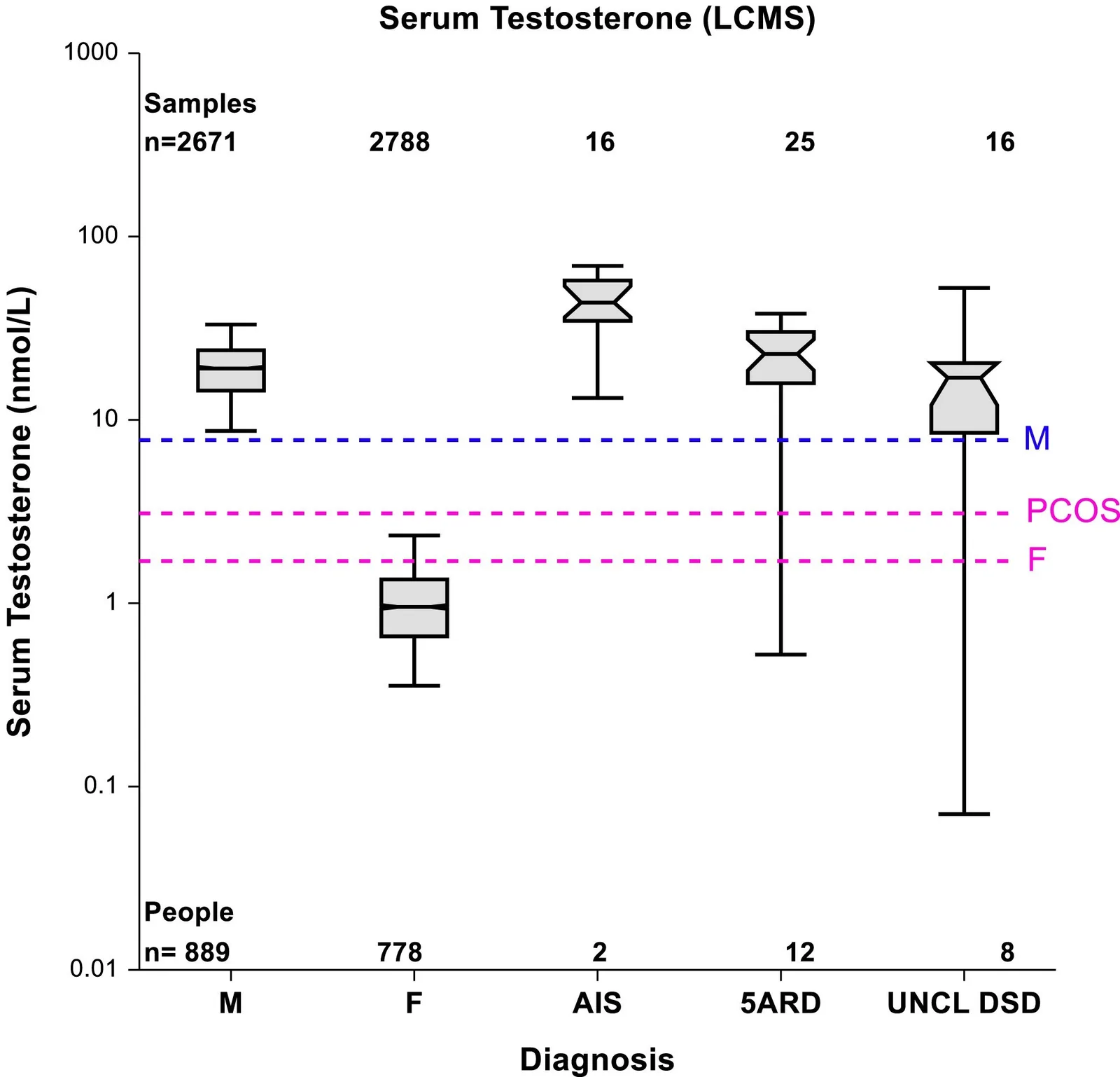
Box and whiskers plot indicating median and quartiles (box) and 95% confidence limits (whiskers) of serum testosterone concentrations (nmol/L) in self-identified male (M) and females (F) with 3 categories of XY DSD identified within the female cohort based on adult male circulating testosterone concentrations. The XY DSDs are separated into androgen insensitivity (AIS), 5α reductase type 2 deficiency, and unclassified XY DSD (UNCL DSD). The numbers represent the number of serum samples (top of panel) and of athletes (bottom of panel). The horizontal lines represent the 95% confidence limit for males (lower 95% confidence limit, blue) and for females (upper 95% confidence limits, pink) without (F) or with polycystic ovarian syndrome (PCOS). The serum testosterone concentrations in nmol/L can be converted to ng/dL by multiplying by 0.345.

**Table 1:** Serum androgens and ratios in elite athletes

N mean ± SEM	Serum T	Serum DHT	Serum A4	Serum T/DHT	Serum T/A4
Male	2671 19.7 ± 0.15 ^f^	1894 1.6 ± 0.02 ^f^	2586 2.19 ± 0.02 ^f^	1903 14.4 ± 0.3 ^f^	2586 10.2 ± 0.1 ^f^
Female	2788 1.1 ± 0.01 ^m^	1804 0.42 0.01 ^m^	2667 3.43 ± 0.04 ^m^	1824 3.9 ± 0.1 ^m^	2666 0.34 ± 0.0 ^m^
5ARD	25 21.6 ± 2.1 ^f^	16 0.64 ± 0.09 ^m^	19 2.69 ± 0.28	16 30.7 ± 4.5 ^f, m^	19 7.6 ± 0.8 ^f, m^
AIS	16 44.3 ± 4.2 ^f, m^	15 2.09 0.20 ^f^	15 3.9 ± 0.39	14 22.6 ± 0.9 ^f, m^	14 12.4 ± 0.6 ^f, m^
UNCL	16 17.1 ± 3.3 ^f^	13 0.39 0.07 ^m^	15 3.59 ± 1.30 ^m^	13 36.8 ± 6.9 ^f, m^	15 6.1 ± 1.0 ^f, m^

All steroid concentrations are in nmol/L. To convert to ng/dL, multiply T by 0.345, DHT by 0.347, and A4 by 0.349.

Superscripts **^m^** and **^f^** indicate the data was significantly different from male and female, respectively, in repeated measures linear mixed model analysis after Bonferroni adjustment for multiple comparisons.

Serum DHT was measured in 3742 samples with 971 reported as undetectable, for which data imputation based on that assay’s limit of detection was used (20). Serum DHT was considered missing where it was reported as not measured.

Abbreviations: 5ARD, 5α-reductase type 2 deficiency; A4, androstenedione; AIS, androgen insensitivity syndrome; DHT, dihydrotestosterone; T, testosterone; UNCL, unclassified.

Table 1 presents these data, excluding samples from pregnant athletes (n = 15) or antidoping rule violations (n = 28, from urine antidoping tests). The serum T, DHT, and A4 and their ratios in the UNCL group most closely resembled those of the 5ARD group (Fig. 2).

**Figure 2:**
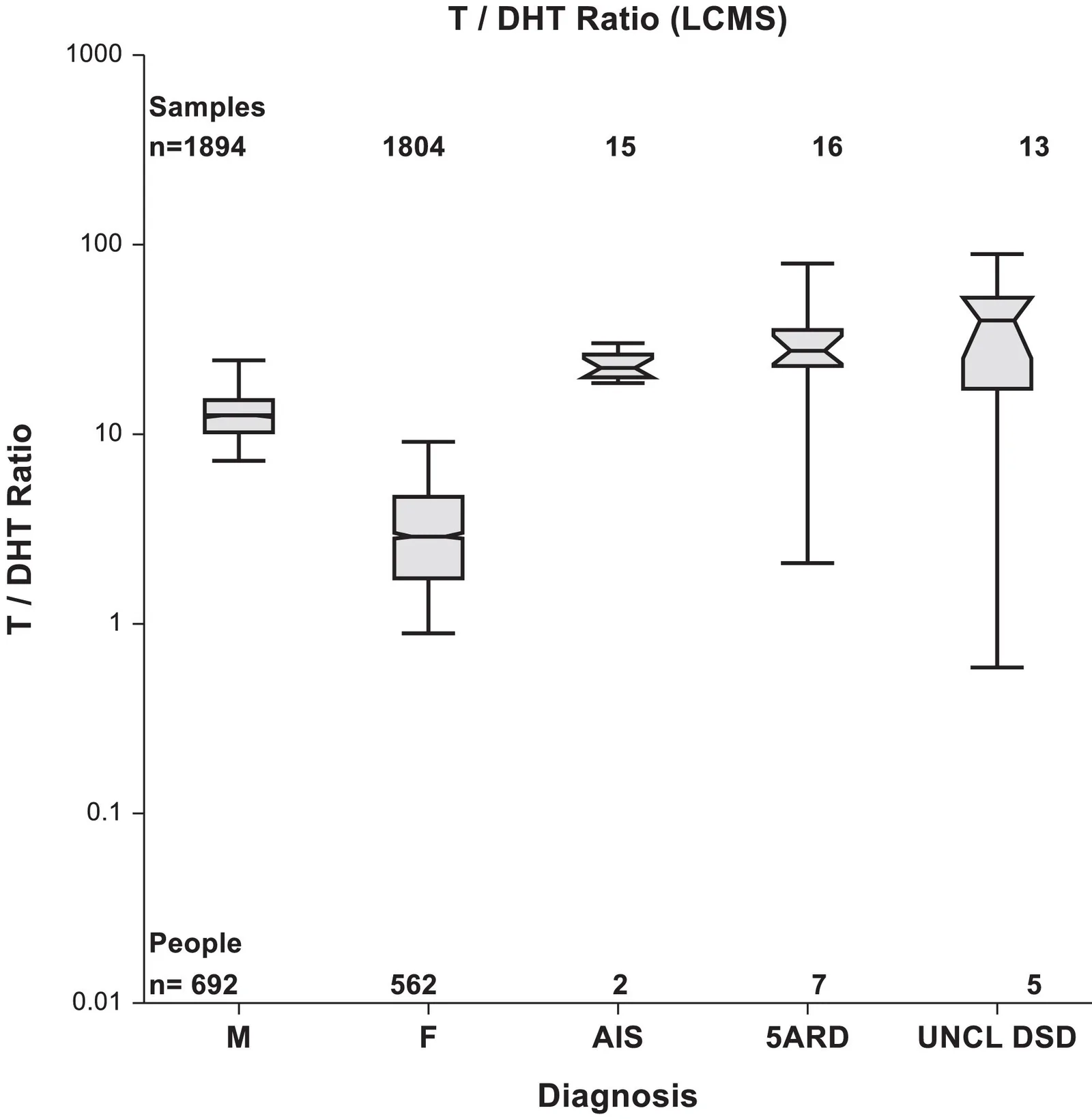
Box and whiskers plot indicating median and quartiles (box) and 95% confidence limits (whiskers) of testosterone to dihydrotestosterone (T/DHT) ratio in self-identified male (M) and females (F) with 3 categories of XY DSD identified within the female cohort based on adult male circulating testosterone concentrations. The XY DSDs are separated into androgen insensitivity (AIS), 5α reductase type 2 deficiency, and unclassified XY DSD (UNCL DSD). The number of serum samples (top of panel) and of athletes (bottom of panel).

The male and female 95% and 90% confidence limits together with median and interquartile range of the T/DHT and T/A4 ratios are shown in Table 2.

**Table 2:** Sex steroid ratios in men and women

	T/A4 ratio	T/DHT ratio	
**Female*^a^***			
N	2666	1804	
95% confidence limits	0.17-0.63	0.7-11.2	
90% confidence limits	0.19-0.55	0.89-9.1	
Median, interquartile range	0.31, 0.25-0.39	2.9, 1.8-4.6	
**Male**			
N	2586	1894	608*^b^*
95% confidence limits	3.2-20.3	6.6-52.2	8.1-30.4
90% confidence limits	4.1-18.1	7.3-24.5	9.0-25.3
Median, interquartile range	9.7, 7.1-12.7	12.5, 10.2-15.0	14.0, 11.6-17.2

Abbreviations: A4, androstenedione; DHT, dihydrotestosterone; T, testosterone.

^*a*^Female excluding known XY DSD individuals.

^*b*^Data from Raine birth cohort (17).

The T/DHT ratios were highly right-skewed with an appearance of at least bimodality (Fig. 3). Analysis of multimodality resolved the data best into 3 peaks with the Bayesian Information Criterion lowest for 3 vs 2 peaks and the dip test rejecting unimodality. The main peak had a mean of 11.1 representing the male population, a secondary peak with a mean of 2.4 represented the female population, with a third peak reflecting 5ARD with a mean of 43.8. The identification of the male and female peaks was confirmed by the same analyses confined to either sex, showing a unimodal distribution with a negative Hartigan dip test featuring a similar expected mean T/DHT ratio.

**Figure 3:**
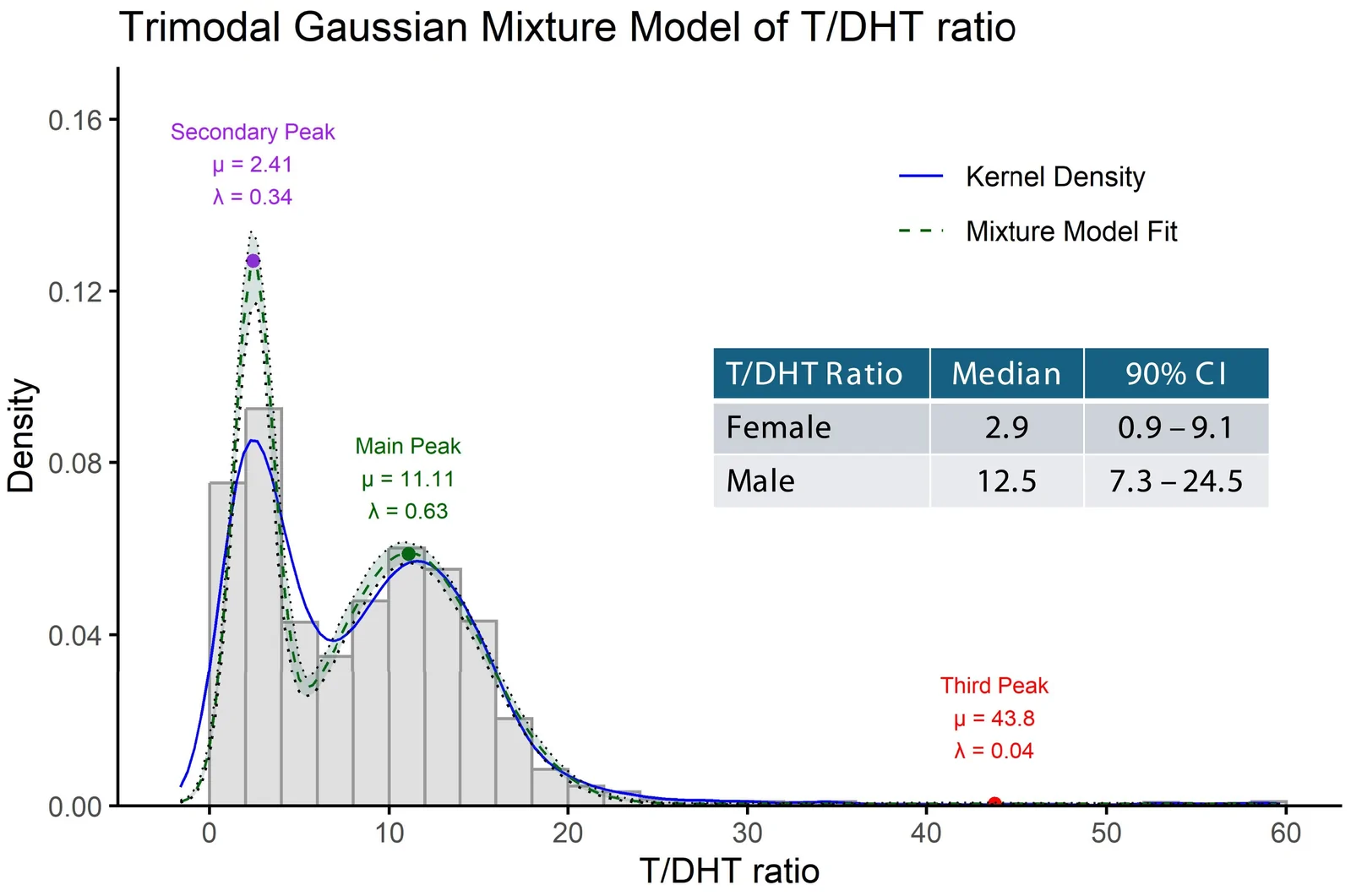
Trimodal Gaussian mixture model plot of T/DHT ratio with data modeled as a kernel density (solid line) and mixture model plot (dashed line) displaying the main (male) peak, a secondary (female) peak, and a third (5α reductase deficiency type 2) peak with their mean T/DHT ratio and weighted proportions (λ).

## Discussion

In the present study of high-performance elite athletes in the AIU RTP, the prevalence of XY DSD was 22/800 (2.8%) among self-identifying female athletes or 22/1689 (1.3%) of all athletes. These proportions may be compared with the best available estimate of the community prevalence of XY DSD, AIS, and gonadal dysgenesis of 6.4, 4.1, and 1.2 per 100 000, respectively (10), representing at least a 200-fold greater prevalence of XY DSD among athletes in this AIU testing pool study compared with the general community prevalence.

A greatly increased prevalence of XY DSD in athletics is consistent with data reported previously for 3 Olympics prior to 2000 (23, 24) and again in 2014 from an IAAF World Championships (25). In these studies, all male and female participants were tested, whereas in the current study a subpopulation selected for high performance was targeted for antidoping testing.

In the community epidemiological prevalence estimates, AIS represents the majority (6.4/11.7 per 100 000, 55%) of XY DSD (10). Yet in this study, 5ARD has much higher prevalence than AIS or CAIS, consistent with androgen-sensitive 5ARD possessing the physical advantages arising from male puberty and thereby being more successful in elite female competition. By contrast, athletes with CAIS are less prevalent, with only one confirmed case, as these individuals experience few, if any, androgenic effects of male puberty and thus have minimal or no male-type physical advantages over typical XX female athletes.

The UNCL group represented those identified as an individual with XY DSD by their male circulating T concentrations, but for whom their final clinical diagnosis was not available. The UNCL group had similar serum T to 5ARD with even lower serum DHT and higher T/DHT ratio than 5ARD, suggesting that a large proportion of the UNCL group also had an unconfirmed 5ARD diagnosis. Hence the prevalence of 5ARD among XY DSD athletes was between 12 to 20 times that of CAIS.

The present interpretation of the prevalence and significance of XY DSD athletes in elite female sports differs from that of Elsas et al (23, 24, 26) who identified XY DSD in Olympic events by SRY gene testing prior to 2000. Those investigators believed those XY DSDs were mostly, if not all, androgen insensitive and therefore such a diagnosis was not an important eligibility criterion because androgen insensitive XY DSD would still be eligible for female events (27). That interpretation of the insignificance of XY DSD for elite female sports led to the discontinuation of SRY gene testing from 2000. Nevertheless, that interpretation is difficult to reconcile with the high prevalence of XY DSD is elite female sports, which suggests unrecognized physical advantages. The present discrepancy between the pre-2000 and present testing is likely to be because the full genetic diagnosis of CAIS now requires identifying a pathogenic inactivating mutation in the androgen receptor (AR) whereas previously the diagnosis of CAIS was based largely or solely on clinical criteria without a proven AR mutation. Such clinical diagnosis of CAIS without confirmatory AR mutation has proven inaccurate in more recent genomic studies, as the clinical features of CAIS overlap with and may be difficult to distinguish from those of other XY DSDs, such as 5ARD and partial AIS or gonadal dysgenesis (11, 28-30), which remain largely androgen sensitive and ineligible for female events under World Athletic eligibility regulations.

This study also provides a large normative dataset of serum T/DHT and T/A4 ratios using LCMS steroid measurements in healthy adult male and female elite athletes compared with prior large sample studies (Table 3). Although the median serum A4 in the present study was very similar to that of the 839 female athletes in the 2011 IAAF World Championships (25), the median serum T was higher than the weighted average of the other 2 female studies (1.1 vs 0.7 nmol/L). That finding is consistent with a subpopulation of XY DSD individuals (median 22.4 nmol/L) with prevalence of 1.8% among the female cohort as well as congruent with the third peak from the trimodal Gaussian mixture model distribution for T/DHT ratio. These data suggesting an included subpopulation of XY DSDs among elite female athletes also may explain why higher serum T concentrations in elite female athletes was positively correlated with competitive performance among elite female athletes (32). Similarly, among male elite athletes, the serum androgen profile was similar to that of the Raine birth cohort study of 618 men aged 20 years, not selected for athletic performance (17). A large study of LCMS-based T/DHT ratio including 4338 men and 2897 women from a non-athletic Swedish population cohort (31), reported similar median T/DHT ratio for men (12.5 vs 12.5) and slightly higher in women (4.2, 2.9) than the present study with narrower interquartile reference ranges for T/DHT for men and women in the present study—again consistent with genetic subpopulations within the self-identified male and female elite athletic populations (Table 2), notably greater proportion of functional polymorphisms of the 5α reductase type 2 enzyme.

**Table 3:** Serum androgen profiles using LCMS from previous studies*^b^*

Study	N	Serum T (nmol/L)*^a^*	Serum A4 (nmol/L)*^a^*
**Females*^b^***			
Bermon (2014) (25)	839	0.69	3.32
Ohlsson (2025) (31)	561	0.71	
Present study	778	1.10	3.43
			T/DHT ratio
**Males**			
Hart (2015) (17)	618	15.8	14.0
Ohlsson (2025) (31)	4338	15.8	12.5
Present study	1894	19.2	12.5

Abbreviations: A4, androstenedione; LCMS, liquid chromatography–mass spectrometry; T, testosterone.

^*a*^Data are medians.

^*b*^Females include young (premenopausal) women excluding pregnant and proven doping.

These reference ranges for healthy young adults may prove useful for the clinical hormonal diagnosis of 5ARD or 17β hydroxysteroid dehydrogenase type 3 (17βHSD3) deficiencies of postpubertal individuals. Although modern standard of care for XY DSD individuals emphasizes early genetic diagnosis, aiming for optimal guidance on appropriate sex of upbringing (11, 12, 30). For individuals with 5ARD or 17βHSD3, the usual recommendation is for a male upbringing due to the prenatal androgen imprinting in an androgen-sensitive individual (12, 33) which avoids disruptive spontaneous switch to male gender in adolescence due to prepubertal brain androgen imprinting (34-36). However, although genetic diagnosis of XY DSD is critical for children with ambiguous genitalia, it is not always readily available for urgent clinical management (11, 35). These hormonal criteria, based on accurate LCMS measurements rather than immunoassays, may provide more rapid guidance to identify the major causes of XY DSD while awaiting full genetic diagnosis. Previously the T/DHT ratio based on steroid immunoassays has demonstrated inaccuracies due to limitations on sensitivity and specificity of T and DHT immunoassays (Table 3), reducing the diagnostic utility of the T/DHT ratio (37), worst for DHT immunoassays which are not standardized. Circulating DHT is produced in androgen target tissues from 2 of 3 functional 5α reductase enzymes (38, 39), although negligibly from the prostate or liver (40, 41). However, only mutation of the type 2 enzyme cause XY DSD and even in individuals with severe genetic deficiency of the type 2 enzyme (5ARD), the type 1 5α reductase enzyme remains active in many tissues (skin, liver, kidney (42)) as well as gut microbiota (31), which reduce the specificity of the T/DHT ratio even when accurately measured by LCMS. The recognized limitations of T and DHT measurements in prepubertal children have led to use of human chorionic gonadotropin (hCG) stimulation in prepubertal children; however, it is not clear how accurately short-term hCG-stimulated T and DHT concentrations reflect 5α reductase type 2 enzyme activity (43) or correspond to those prevailing naturally in postpubertal adults.

The utility of the A4/T ratio for diagnosis of 17β HSD3 deficiency is less clear, as this condition is even rarer than 5ARD and AIS (44, 45). Although individuals with 17βHSD3 deficiency (44, 46) are androgen sensitive with distinctive hormonal biochemistry, they are clinically very similar to 5ARD (47), and the present analysis has not identified such individuals presumably due to their greater rarity than the relatively more frequent forms of XY DSD. The 17βHSD type 3 enzyme is expressed solely in the testis, and its genetic deficiency can lead to ambiguous or female appearing genital in genetic male neonates and infants; however, at puberty or later the excess circulating A4 can be converted to T by other 17βHSD enzymes among at least 14 variants reported (48). For adults the A4/T ratio exceeding 0.31 would support a diagnosis of 17βHSD3 deficiency; however, in this dataset only 15/889 (1.7%) exceeded that upper 95% threshold and are likely just the extreme ends of that distribution. No female athlete had a A4/T ratio exceeding 0.31 with T also exceeding the female range while only a single male athlete met these criteria raising the possibility of a presumptive diagnosis of 17BHSD3; however, that individual, self-classified as a male, would not breach eligibility regulations for female events.

We conclude that most XY DSD individuals entering in elite female athletics events have 5ARD and are androgen sensitive. Using LCMS measurements, 5ARD and UNCL have similar T/DHT ratio and serum T concentrations to males, suggesting many if not most of the UNCL individuals also have 5ARD, which then has 12 to 20 times the prevalence of CAIS among XY DSD athletes. The serum T/DHT ratio (by LCMS) can extend to characterize 5ARD in not just sedentary individuals but also among elite athletes.

## Disclosures

S.B. is an employee of World Athletics. D.J.H. has been and is a member of World Anti-Doping Agency advisory committees.

## Data Availability

Some or all datasets generated during and/or analyzed during the current study are not publicly available but are available from the corresponding author on reasonable request

## Abbreviations

5ARD: 5α-reductase type 2 deficiency
17βHSD3: 17β hydroxysteroid dehydrogenase type 3
A4: androstenedione
AIS: androgen insensitivity syndrome
AIU: Athletics Integrity Unit
AR: androgen receptor
CAIS: complete androgen insensitivity syndrome
DHT: dihydrotestosterone
DSD: disorders of sex development
LCMS: liquid chromatography–mass spectrometry
RTP: Registered Testing Pool
T: testosterone
UNCL: unclassified
WADA: World Anti-Doping Agency
